# Inequalities in local government expenditure on environmental and regulatory services in England from 2009 to 2020: a longitudinal ecological study

**DOI:** 10.1136/bmjph-2024-001144

**Published:** 2024-12-04

**Authors:** Lauren Murrell, Katie Fahy, Helen E Clough, Roger Gibb, Xingna Zhang, Marie Anne Chattaway, Mark Alan Green, Iain Edward Buchan, Ben Barr, Daniel Hungerford

**Affiliations:** 1 National Institute for Health and Care Research Health Protection Research Unit in Gastrointestinal Infections, University of Liverpool, Liverpool, UK; 2 Department of Clinical Infection, Microbiology and Immunology, University of Liverpool Institute of Infection Veterinary and Ecological Sciences, Liverpool, UK; 3 Department of Public Health, Policy & Systems, University of Liverpool Institute of Population Health, Liverpool, UK; 4 Department of Livestock and One Health, University of Liverpool Institute of Infection Veterinary and Ecological Sciences, Liverpool, UK; 5 Public Contributor, PPI Advisor to: Health Protection Research Unit Gastrointestinal Infection, University of Liverpool, Liverpool, UK; 6 Gastrointestinal Bacteria Refence Unit, United Kingdom Health Security Agency (UKHSA), London, UK; 7 NIHR Health Protection Research Unit in Genomics and Enabling Data, University of Warwick, Warwick, UK; 8 Geography & Planning, University of Liverpool, Liverpool, UK

**Keywords:** Epidemiology, Public Health, Food Services, Public Health Practice

## Abstract

**Background:**

Gastrointestinal (GI) infections affect one in five people in the UK and local authorities play a crucial role in controlling these infections. However, there have been substantial reductions in funding for environmental and regulatory (ER) services that enable GI infectious disease prevention and control via food safety and infection control (FSIC) services. This study investigates how local funding cuts to these services have varied across England to understand the potential consequences of inequalities in GI infections.

**Methods:**

We carried out a longitudinal observational ecological study, using a panel of annual data between 2009/2010 and 2020/2021. Analysis of ER service expenditure and FSIC service expenditure included 312 and 303 local authorities respectively. Generalised estimating equation models were used to estimate the annual per cent change of ER service expenditure between 2009/2010 and 2020/2021 in addition to FSIC expenditure change overall, and as a share of total ER expenditure. Models analysed trends by local authority structure, population density and deprivation level.

**Results:**

ER services saw the largest cuts in unitary authorities, declining by 1.9%. London boroughs had the greatest reductions in FSIC expenditure, decreasing by 9.9%. Both ER and FSIC expenditure decreased with increasing population density. Areas of higher deprivation had the largest reduction in expenditure, with ER and FSIC cuts of 2.4% and 22.8%, respectively, compared with a 1.2% and 7.5% reduction in the least deprived areas. The share of ER expenditure spent on FSIC decreased by 13.4% in the most deprived authorities compared with 6.3% in the least deprived areas.

**Conclusion:**

The unequal distribution of cuts shows the need for increased and equitable investment into these services to enable resilience to emerging infectious disease threats and to prevent the widening of health inequalities.

**WHAT IS ALREADY KNOWN ON THIS TOPIC**

- Austerity measures have led to substantial reductions in local funding, research shows reductions vary by deprivation level of an area, rural–urban classification and local authority structure.
- It is unknown how local funding cuts to environmental and regulatory services, which provide essential services for public health protection, vary by these characteristics.

**WHAT THIS STUDY ADDS**

- We investigate inequalities in changes to environmental and regulatory service expenditure and subspending lines that cover food safety and infection control functions during the time of austerity by local authority structure and population density and deprivation from 2009/2010 to 2020/2021.
- The largest cuts were in the more deprived areas and with increased population density for both environmental and regulatory and food safety and infection control services. The largest cuts in environmental and regulatory services were seen in unitary authorities whereas food safety and infection control services saw the largest cuts in London boroughs.

**HOW THIS STUDY MIGHT AFFECT RESEARCH, PRACTICE OR POLICY**

- This research provides strong evidence of inequalities in local authority service expenditure in environmental and regulatory services and highlights where investment should be focused, in order to protect environmental and public health.

## Background

 Over the past 10 years, the UK has faced substantial changes to public spending at the local authority level. Following the financial crisis of 2008, austerity measures were implemented to reduce fiscal deficit and stabilise the economy.^1^ These measures led to significant cuts to local authority funding across services, which saw central government funding decrease by 50% between 2010/11 and 2017/18.^2 3^ Local authorities are responsible for providing key services such as social care services, housing, leisure and cultural services, public health and environmental and regulatory (ER) services (sometimes referred to as environmental health or regulatory services).^4^ ER services include functions which are key in public health protection. These are separate from public health services which include sexual health services, treatment and prevention of drug and alcohol misuse, and public mental health.^5^ These services are generally carried out by a separate department from ER services. A growing body of literature describes the considerable impact of local funding cuts, demonstrating disproportionately grave impacts on disadvantaged areas,^6^,^8^ including increased disease burden.^6^

Established in 1848, the UK Public Health Act empowers local authorities to protect the health of the population, addressing issues like infectious diseases, water safety and waste management.^9^ Local authorities also work together with other bodies such as the UK Health Security Agency (UKHSA) in disease outbreak control,^10^ such as during COVID-19, when local response was distributed among local authorities, the UKHSA and other services. ER services align with the mandates of the Public Health Act, delivering water safety, waste collection, trading standards, food safety and infectious disease control.^11^ These services allow investment in the safeguarding of local public health, with ER services responsible for the notification and prevention of infectious disease.^12^ In 2020, ER services dealt with 58 434 notifiable incidents of infectious disease, with 4800 cases due to food poisoning.^9^ Food safety services within ER are responsible for regular food hygiene inspections, testing of food samples, provision of advice and education material and investigation of foodborne disease outbreaks and illness.^11^ Meanwhile, service expenditure under Infection Control services is focused on infectious disease control functions under the Public Health Act of 1984 and the associated 1988 regulations.^11^ Functions and regulations specified under this act include the reporting, management and control of infectious diseases.^13^

Gastrointestinal (GI) infections are a group of diseases that are transmitted through contaminated food and water, the environment and person to person.^14^ They are a significant public health concern in the UK, with approximately 17 million cases annually.^15^ These illnesses impact people in more deprived communities the most, these communities experience more severe outcomes^16^ such as hospitalisation^17^ and higher risk of infection.^18^ Food safety and infection control services (FSIC) are crucial in prevention and control of GI illness, carrying out activities such as hygiene inspections of food establishments, and case finding and follow-up of notifiable GI infections. Therefore, local funding cuts to these services may increase the risk of GI infection and illness and worsen existing health inequalities.

Local authority funding comes from a combination of central government grants, local council tax, income from fees and charges. National government decides on the overall central government grants for local authorities. The government then decides what expenditure cuts are made to central government grants. These cuts disproportionately affect the more deprived authorities,^19 20^ which rely on this funding more.^20^ The distribution of this budget to services is decided by the local government and differs by their structure and responsibilities. Here local government is partially constrained by the statutory or discretionary nature of services. Statutory services, such as some provided by ER services, will be prioritised over more discretionary services. Unitary authorities, including London boroughs, are single-tier authorities and are responsible for all functions.^21^ Two-tier authorities split responsibilities between the upper (county) and lower (district) levels. As single-tier authorities cover all local government responsibilities, they have more scope to redirect funding from more discretionary service budgets in order to protect statutory services in particular social care, which is by far one of the largest areas of statutory local authority expenditure.^21^ However, two-tier authorities are limited in their ability to redirect funding from lower-tier services, such as ER services, to upper-tier services such as social care. Therefore, the resulting spend in a place follow from an interaction of these factors. The other influence on how local authority funding is shared between local authorities and between service areas within local authorities is the assessment of need. The central government grant is weighted based on an overall assessment of need,^22^ and local prioritisation between service areas will reflect local assessments of needs in council budget-setting processes. Deprivation, population structure and population density are key drivers of need for ER services. Differential reductions in ER service spending over this time period will, therefore, relate to the interplay of these factors, including national decision-making on the overall budget, organisational mechanisms such as local government structure, and drivers of need and income, such as population density and deprivation.

ER services have received substantial cuts since the introduction of austerity measures.^2 23 24^ These spending cuts have been accompanied by service changes, including reports of reductions in Food Safety staff by 13% per 1000 food businesses between 2012/2013 and 2017/2018,^25^ a decline in food standards and hygiene sampling and decreased waste removal.^3 25 26^ Existing research indicates that even before austerity, ER services were insufficient to meet the needs of the most deprived neighbourhoods.^27 28^ Furthermore, environmental health teams reported their capacity to function during the COVID-19 pandemic was impacted by prior local funding cuts.^29^ Therefore, expenditure reductions to these services may further exacerbate inequalities in service provision and widen inequalities in GI infection-associated health outcomes.

This study aims to describe trends in ER service expenditure since the introduction of austerity. In addition, we investigate spending lines of ER services we believe are instrumental in the prevention and control of GI infectious disease, FSIC services. We investigate how expenditure has differed by local authority structure, urbanicity service need and level of neighbourhood socioeconomic deprivation. This is the first in a series of studies that sets out to understand the potential impact of local funding cuts on ER services and outcomes of GI infections.

## Methods

The study protocol containing further details of the study design, the analytical framework, data sources and indicators have been previously published.^30^ This protocol describes the aims and objectives of a larger project, here we report the methods and results pertaining to objective one of the protocol, the first results section of the overall project. We present a concise summary of the methods below.

### Setting

We analysed annual expenditure data in England from 2009/2010 to 2020/2021 for lower-tier and single-tier local authorities. The geographical boundaries used in this analysis are based on 2020 local authority boundaries. The City of London and Isles of Scilly were excluded from the study due to their small populations and unusual funding and health infrastructure, leaving 312 local authorities for ER service expenditure analysis. Additionally, for the analysis of FSIC, a further nine local authorities were removed due to missing data, leaving 303 authorities for analysis.

### Data and measures of interest

We define two measures indicating expenditure available for activities related to GI infection control. First, we look at total gross ER expenditure, recorded under cultural, environmental, regulatory and planning services in local authority out-turns,^11^ revenue out-turn 5. Second, we derived a measure of funding more specifically available for FSIC by aggregating expenditure lines recorded under ER services, the spending lines of food safety and animal and public health infectious disease control. Our discussions with environmental health officers indicated that these spending lines covered costs for staff responsible for FSIC. These staff may also have other responsibilities not directly relevant to infection control such as animal welfare and dog control and overseeing traveller sites,^11^ but a substantial part of their role is FSIC. We provide further information on ER spending lines and a full list of services included under FSIC spending lines in online supplemental material (page 1). Input from environmental health officers indicated that cross-reporting may occur, meaning expenditure on infection control services may be reported under the food safety spending line. This insight informed our decision to aggregate these spending lines, as there may not be sufficient granularity across all local authorities to analyse these budgets separately.

We use expenditure data from official budget out-turns that local authorities are required to report to government,^31^ compiled by the Place-Based Longitudinal Data Resource.^32^ Data are provided at the lower-tier local authority level each year from 2009/2010 to 2020/2021. Gross expenditure is defined as the total spending by local authorities, including income raised in service provision, data are indicative of the financial year—1 April to 31 March. The midyear population estimate for each local authority was obtained from the Office for National Statistics (ONS) to estimate per capita expenditure. Expenditure data were adjusted for inflation prior to analysis using gross domestic product deflator.^33^

Data on local authority structure were obtained from the ONS.^34^ The structure of a local authority is a key factor that might explain the difference in expenditure reductions to ER services. Local authorities were grouped into three categories, London boroughs, two-tier or unitary (including metropolitan) authorities.

Population density estimates were obtained from ONS.^34^ Population density may influence infectious disease risk due to greater transmission in urban areas. Additionally, it acts as an indicator of food outlet density. Here, we use population density to look at urbanicity and level of service need. Population density estimates were originally measured in persons per square kilometre, in this study population, density was divided by 1000, so that a unit increase corresponded to a change of 1000 persons per square kilometre. This facilitates interpretation of model estimates as a unit of one person per square kilometre is less meaningful.

English Indices of Multiple Deprivation (IMD) were used as the measure of deprivation. Data on IMD were obtained from the Ministry of Housing, Communities and Local Government.^35^ We analyse trends in ER service expenditure across local authorities of different socioeconomic deprivation to investigate and describe any inequalities present in the distribution of reduction in service expenditure. Local authorities were assigned to their deprivation quintile using IMD 2019 data. The local authority IMD average score was used to produce quintiles weighted by population size, ensuring the number of people in each quintile evenly distributed.

### Analysis

Data were analysed at the aggregated ER service level (online supplemental figure S1). Then ER and FSIC service data were descriptively explored, investigating how expenditure changed over time by deprivation, population density (classed as predominantly rural or predominantly urban) and by local authority structure in exploratory analysis. Categories for predominantly rural and predominantly urban were based on the Rural Urban Classification from the Department for Environment, Food and Rural Affairs.^36^ Here, we include local authorities that are Urban with Significant Rural (defined as between 26% and 49% of the population resides in rural areas) as predominantly urban. We indexed values from 2009 to show a change in expenditure over time, in addition to presenting raw spending figures (online supplemental figures S2 and S3).

To measure temporal trends in expenditure, data were stratified by deprivation level, local authority structure and population density. We used a generalised estimating equation (GEE), which allows us to analyse the independent relationships between expenditure and each local characteristic. For example, this model allows us to understand the relationship between expenditure and local authority structure when we hold deprivation constant. GEEs are commonly used in longitudinal analysis to estimate average population effects. We use a log link function to assess temporal trends in expenditure and explanatory variables.^37^ The outcome was annual gross total ER service expenditure, with log-transformed population size as an offset, in order to model the log of per capita spend. Financial year was specified to interact with local authority structure, population density and deprivation level. This model was then reproduced for gross FSIC service expenditure. The proportion of FSIC expenditure as a share of total gross ER service expenditure was explored, with FSIC expenditure as the outcome variable and log-transformed gross ER service expenditure as the offset. We interpreted the GEE model results by calculating the linear combinations of the estimated coefficients. The results were presented as annual percentage change with 95% CI.

### Missing data and multiple imputation

Missing data were present across FSIC spending lines (online supplemental table S1). It is possible that misclassification and cross-reporting led to reporting of zero for expenditure. FSIC services had 17 local authorities with missing data present (online supplemental table S2). Of these, nine were removed if data were missing for three consecutive years, two consecutive years at the beginning (2009 and 2010) or at the end of the study period (2019 and 2020) (see online supplemental figure S4). Eight were carried forward for multiple imputation with 10 observations missing, using predictive mean matching and a predictor matrix consisting of deprivation level, local authority type and population data to estimate missing values. A density plot was produced to display the distribution of imputed estimates against observed values (online supplemental figure S5). Sensitivity analysis was carried out with casewise deletion of authorities with missing values (online supplemental table S3).

### Patient and public involvement

In the early stages of this project, a patient and public involvement and engagement (PPIE) panel was held where input from panel members contributed to initial planning. This included an initial discussion of the variables that would be included in the project. Following this, a PPIE member was involved throughout the research, contributing to the study design and conceptualisation, dissemination of this work and coauthored this paper.

## Results

### Trends in expenditure

There was a decline in mean expenditure for ER and FSIC services per capita between 2009/2010 and 2020/2021, across local authority type, rural and urban local authorities, and each deprivation level (table 1). We see that the more deprived authorities and London boroughs tended to have a higher baseline mean expenditure at the beginning of the study period. The most deprived local authorities, London boroughs and urban authorities saw the largest reductions in mean expenditure per capita for ER and FSIC service expenditure.

**Table 1:** Mean expenditure in 2009/2010 and 2020/2021 summary table

	Baseline ER mean expenditure per capita 2009/2010, £ (SD)	Change in mean ER expenditure per capita 2009/2010–2020/2021, £ (%)	Baseline food safety+infection control expenditure per capita 2009/2010, £ (SD)	Change in mean food safety+infection control expenditure per capita 2009/2010–2020/2021, £ (%)	Number of local authorities	Mean population density per square kilometre (SD)
Local authority structure						
London Borough	217.82 (83.88)	−50.37 (−23.10)	10.75 (7.21)	−7.75 (−72.10)	32	7344 (3760)
Two-tier	153.38 (23.92)	−17.26 (−11.30)	9.20 (6.24)	−5.24 (−57.0)	188	769 (923)
Unitary (excluding London)	159.34 (30.15)	−38.00 (−23.80)	7.92 (5.78)	−5.33 (−67.30)	92	1793 (1434)
Rural or urban						
Predominantly rural	154.15 (21.54)	−16.89 (−11.00)	9.59 (6.11)	−5.87 (−61.2)	86	164 (88)
Predominantly urban	164.64 (45.99)	−30.53 (−18.6)	8.75 (6.30)	−5.39 (−61.60)	226	2348 (2734)
IMD quintile						
1 (least deprived)	144.35 (21.52)	−15.02 (−10.40)	8.74 (6.04)	−5.09 (−58.20)	88	581 (820)
2	155.84 (21.08)	−22.74 (−14.60)	8.41 (5.08)	−4.39 (−52.20)	67	1338 (1729)
3	169.24 (60.57)	−24.49 (−14.50)	8.37 (5.39)	−5.28 (−63.10)	68	2035 (2802)
4	176.23 (47.17)	−37.85 (−21.50)	10.84 (8.57)	−7.32 (−67.50)	45	3429 (3779)
5 (most deprived)	179.16 (35.20)	−48.64 (−27.10)	9.37 (6.59)	−6.67 (−71.20)	44	2551 (2435)

ERenvironmental and regulatory

### ER service expenditure

**Figure 1:**
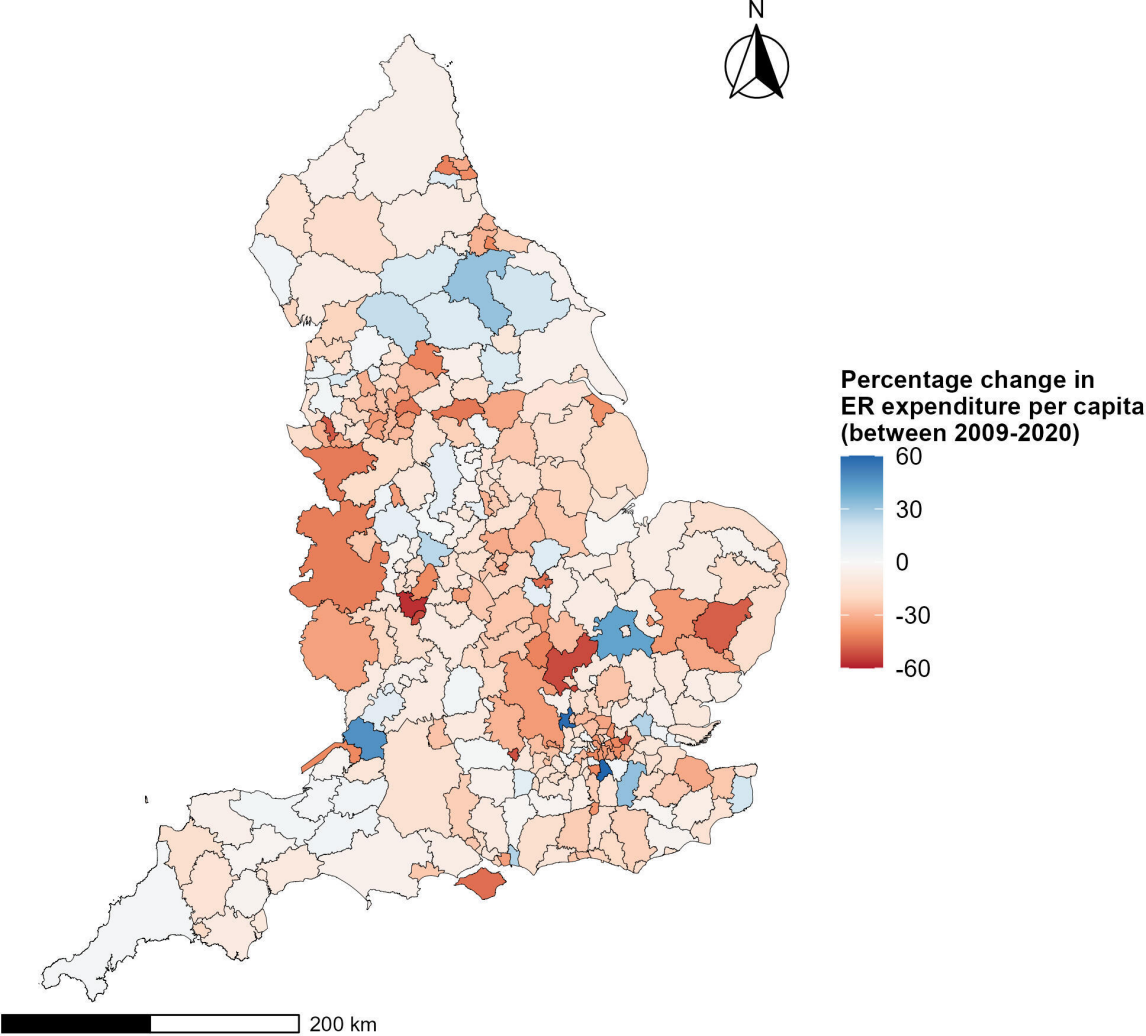
The percentage change in environmental and egulatory (ER) service expenditure between 2009 and 2020 at the local authority level. The shapefile of England is obtained from the Office of National Statistics. Source: Office for National Statistics licensed under the Open Government Licence V.3.0. Contains OS data Crown copyright and database right 2024.^42^

There is variation in the direction and size of expenditure change between local authorities. Most local authorities experienced an overall decline in expenditure, with the largest being 57%. However, 49 local authorities experienced an increase in ER expenditure in 2020/2021 compared with 2009/2010, with the largest being 60% (figure 1).

**Figure 2:**
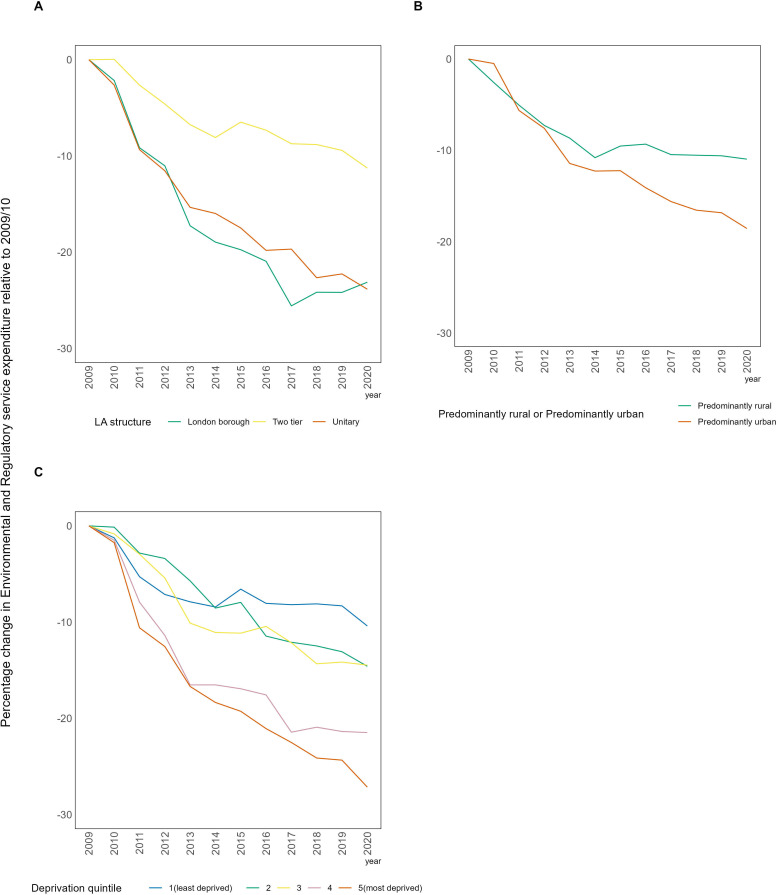
The percentage change in mean ER expenditure per capita relative to 2009/2010, stratified by deprivation, local authority (LA) structure and predominantly rural/urban. ER, environmental and regulatory.

In London boroughs and unitary authorities, expenditure experienced a larger decline in gross ER service expenditure compared with two-tier authorities, which had a maximum reduction of 11% in 2020/2021 (figure 2). By 2012, London boroughs and unitary authorities surpassed this decline, both experiencing reductions of over 23%. During this period, relative cuts to gross ER service expenditure per capita in predominantly urban areas were almost double that in predominantly rural areas. Expenditure per capita decreased by 27% in the most deprived local authorities, from 2009/2010 to 2020/2021, compared with a 10% decrease in the least deprived local authorities.

### FSIC expenditure

**Figure 3:**
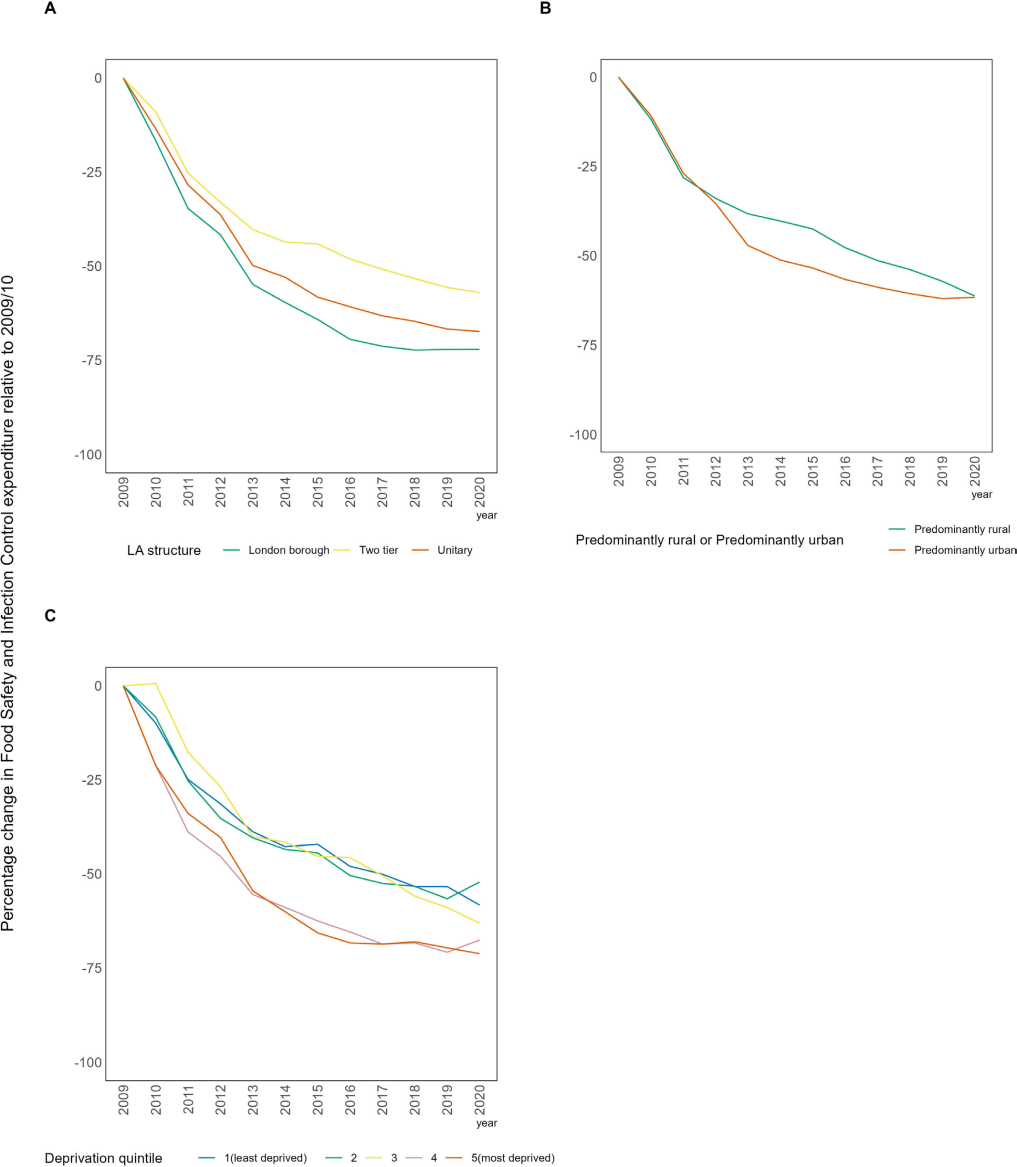
The percentage change in mean Food Safety and Infection Control services expenditure per capita relative to 2009/2010, stratified by deprivation, local authority (LA) structure and predominantly rural/urban.

There was a steep decline in expenditure on FSIC services. London boroughs saw the largest cuts in expenditure over time, with 2020/2021 cut by 72% relative to 2009/2010 (figure 3). Two-tier authorities saw the smallest reduction in expenditure of 57% between 2009/2010 and 2020/2021. Predominantly rural and predominantly urban local authorities had seen a matched decline until 2012, from this point predominantly urban areas saw a larger decline in annual expenditure, until 2020/2021 when both groups experienced a similar decrease of 61.6% and 61.2%. Service expenditure declined by 71% by 2020/2021 in the most deprived local authorities.

### GEE expenditure trends

The estimated trends in ER service expenditure per capita from the GEE models show annual declines in expenditure (table 2). Trends in expenditure also varied between local authority structure. The largest cuts were in unitary authorities, which saw an annual decrease of 1.9% (95% CI −2.9%, −0.8%) in expenditure, compared with a decrease of 1.0% (95% CI −3.0%, 1.0%) in London boroughs. For each additional 1000 people per km^2^, there was a decrease of 1.4% (95% CI 95% CI −2.1%, –0.7%) in expenditure annually. The most deprived local authorities experienced a reduction of 2.4% (95% CI −3.5%, −1.3%) in expenditure annually compared with 1.2% (95% CI −1.8%, −0.5%) in the least deprived local authorities.

**Table 2:** Estimates of annual percentage change in ER, FSIC expenditure per capita from generalised estimating equation models, between 2009/2010 and 2020/2021

Variable	Annual percentage change in ER per capita (95% CI)	Annual percentage change in FSIC expenditure per capita (95% CI)	Annual percentage change in FSIC as a share of ER (95% CI)
LA structure			
Two-tier	−1.2 (−1.8, −0.5)	−7.5 (−9.1, −5.9)	−6.3 (−7.9, −4.7)
Unitary	−1.9 (−2.9 −0.8)	−8.7 (−11.7, −5.7)	−8.4 (−11.6, −5.2)
London Borough	−1.0 (−3.0, 1.0)	−9.9 (−16.4, −2.9)	−9.2 (−16.1, −1.7)
Population density			
Population density	− 1.4 (−2.1, −0.7)	−8.1 (−10.1, −6.0)	−6.4 (−8.4, 0.0)
IMD quintile			
1 (least deprived)	−1.2 (−1.8, −0.5)	−7.5 (−9.1, −5.9)	−6.3 (−7.9, −4.7)
2	−1.3 (−2.0, −0.6)	−8.6 (−12.4, −4.5)	−7.2 (−11.1, −3.2)
3	−0.1 (−1.0, 0.8)	−5.8 (−9.2, −2.2)	−5.1 (−9.1, −0.9)
4	−1.3 (−2.2, −0.4)	−9.4 (−13.8, −4.8)	−6.7 (−11.5, −1.7)
5 (most deprived)	−2.4 (−3.5, −1.3)	−22.8 (−34.9, −8.4)	−13.4 (−21.1, −5.0)

ERenvironmental and regulatoryFSICFood Safety and Infection ControlIMDIndices of Multiple DeprivationLAlocal authority

Of local authority structures, London boroughs experienced the largest reduction in FSIC expenditure with a decrease in expenditure per capita of 9.9% (95% CI −16.4%, −2.9%). There was a decrease in FSIC expenditure of 8.1% (95% CI −10.1%, −6.0%) annually for each additional 1000 people per km^2^ increase in population density. The greatest decrease in FSIC expenditure was in the most deprived local authorities (−22.8%, 95% CI −34.9%, −8.4%) annually compared with the least deprived (−7.5%, 95% CI −9.1%, −5.9%).

The share of ER service expenditure spent on FSIC was cut the most in London boroughs (−9.2%, –95% CI −16.1%, −1.7%) and decreased by 6.4% (95% CI −8.4%, 0%) annually, per 1000 increase in population density. The share of expenditure spent on FSIC decreased by 13.4% (95% CI −21.1%, −5%) annually in the most deprived authorities compared with 6.3% (95% CI −7.9%, −4.7%) in the least deprived.

### Sensitivity analysis

We found that the results of the analysis, excluding local authorities with zero expenditure data for FSIC, were similar to those from the multiple imputation analysis. For further details, refer to online supplemental table S3.

## Discussion

### Summary

In this longitudinal study, we investigate inequalities in ER service expenditure and spending lines of FSIC during a time of austerity. Our study demonstrates how austerity has exacerbated social and spatial inequalities in environmental public health, which could lead to widening health inequalities. Expenditure for total ER services decreased more in unitary local authorities, local authorities with higher population density and in the most socioeconomically deprived local authorities. Expenditure for FSIC decreased most in London boroughs and unitary authorities compared with two-tier authorities which saw the smallest reductions, overall and as a share of ER service expenditure. Furthermore, overall FSIC expenditure decreased in areas with higher population density, as did the share of ER service expenditure spent on FSIC. These inequalities in expenditure cuts are accentuated when analysing FSIC, with the most deprived areas experiencing the largest reductions over time and as a share of total ER service expenditure.

### Interpretation

Our study found the largest reductions in ER service expenditure to be in unitary authorities, while London boroughs and unitary authorities saw the largest reductions in FSIC expenditure, overall and as a share of ER service expenditure. This is in line with previous research by Fahy *et al* which describes the largest reduction in cultural, environmental and planning (CEP) in unitary authorities.^38^ This is likely due to single-tier authorities making larger cuts to and within the ER service budget to protect higher priority services, such as social care and is exacerbated by the different capacities to subsidise services with alternative revenues.^24^ For instance, in 2018/2019, unitary local authorities generated 15% of spending needed from fees and charges compared with 60% in district councils.^39^ Furthermore, unitary authorities raise less revenue from tax income compared with county councils,^26^ which reflects the difference in ability to generate revenue.

Findings showed that as population density increased, so did cuts in overall ER service expenditure, FSIC and FSIC as a share of ER service expenditure. This is in line with previous research that showed urban areas in England have faced more substantial reductions in CEP spending compared with rural.^38^ Budgets in areas with higher population density may see larger cuts due to larger reliance on central government funding in urban areas compared with rural areas, which have alternative means of income generation.^2^ This is of concern as areas with higher population density are likely to have higher food establishment density, increasing the service need in these areas.

Reductions in expenditure across ER services and within FSIC were largest in the most deprived local authorities compared with the least deprived, supporting previous findings on expenditure cuts.^8 38^ These reductions were larger for FSIC services, with annual decreases of 22.8% compared with 2.4% for overall ER. This suggests a variation between the subservices, which might be missed in higher-level analysis. A report by the National Audit Office describes differences in spending between ER subservices, with community safety falling by 47.1% between 2010/2011 and 2015/2014 compared with 11.7% for waste services during the same period,^26^ highlighting the importance of subservice level analysis. Statutory services such as those carried out by ER services have seen smaller reductions compared with other services.^3 26^ However, local authorities facing larger cuts are less able to protect these services from reductions.^26^ While ER services are largely statutory, meaning that local authorities must legally provide them, national legislation does not require a certain level of funding or quality. This makes them vulnerable to funding cuts compared with protected services, like social care which must be prioritised for delivery and are safeguarded against cuts. Therefore, we expect the increasing demand for social care^3^ would result in greater cuts to ER indirectly which may impact inequalities. In addition, more deprived local authorities may struggle to generate income outside of central government funding (their primary source of funding), through means such council tax and business rates due to factors such as lower property values and lower business rates.^2^

Inequitable cuts to statutory service funding will result in inequitable service delivery and impact. Research shows that food establishments in the most deprived areas are 25% less likely to obtain required hygiene standards than those in the least deprived areas.^40^ Furthermore, individuals in more deprived areas are at greater risk of GI infections^18^ and experience more severe outcomes.^17 41^ This inequality in infection risk is particularly true for pathogens associated with person-to-person transmission and some foodborne pathogens.^18^ These trends may reflect potential implications of unequal funding cuts to these services and are of particular concern, as more deprived areas tend to have a higher demand for FSIC services but have experienced the largest reduction in funding. Ultimately, these cuts may see a reduction in the number of inspections achieved, an increase in unsafe food environments, and a decline in staff and resources available for service delivery. We may also see a limited capacity for outbreak management, as exemplified in some environmental health departments during COVID-19.^29^ Therefore, these cuts could place the public at an increased risk of GI infection and illness.

### Strengths and limitations

This study is the first to analyse expenditure of ER services using statistical methods, spatially and temporally. We use national data for these, including the analysis of critical subservices, while accounting for population change and inflation, providing a comprehensive indication of trends in expenditure over time. This provides evidence, which may be used in informing policy and direction of needs at a larger scale. Study limitations include uncertainty surrounding subservice reporting accuracy and the missing data within these services. It is possible that misclassification and reporting errors occurred within the reporting of local authority expenditure resulting in some subservices reporting zero expenditure. Furthermore, the difference in missingness may be due to the discretionary characteristics of a service, meaning they are not prioritised, and funding may be directed elsewhere. We tried to account for this by combining subservices of interest, in addition to using multiple imputation. Another limitation is the use of population density as a proxy for food establishment density. However, the quality of the population density data made it a more suitable measure. Additionally, this analysis does not delineate geographical characteristics which might impact ER expenditure, such as if a local authority is coastal or inland which could be an area of future study. Finally, this study is descriptive, therefore, we cannot confirm why these trends have occurred or what the implications of these findings might be, this should be investigated in future work.

## Conclusion

Despite the importance of ER services as a key component of local health protection systems, they have been cut substantially year-on-year since the introduction of austerity. The concerning trend that cuts to these protective services have hit more deprived, highly populated urban areas, presents a high risk for resilience to current and future infectious disease threats. This observed decline is unsustainable and threatens the ability of this service to function, posing a potential threat to the public from infectious diseases. Further research is needed to understand the impact on health outcomes of these inequitable cuts to ER services. Additionally, increased and equitable investment in these services is urgently needed to protect public health.

## supplementary material

10.1136/bmjph-2024-001144 online supplemental file 1

## Data Availability

Data are available in a public, open access repository.
