# Supplementary material for: Inequalities in local government expenditure on environmental and regulatory services in England from 2009 to 2020: a longitudinal ecological study
Source: BMJ Public Health. 2024 Dec 4;2(2):e001144. doi: 10.1136/bmjph-2024-001144 (PMC11816392; doi:10.1136/bmjph-2024-001144)
Supplement: online supplemental file 1 [file bmjph-2-2-s001.docx]

# Supplemental material

## Environmental and Regulatory service spending lines

### Environmental and Regulatory service spending lines

- Food safety gross
- Water safety gross
- Trading standards gross
- Port health gross
- Public conveniences gross
- Animal and Public health infectious disease control (Infection Control) gross
- Pest control gross
- Environmental protection noise and nuisance gross
- Defence agianst flooding
- Land drainage and related work
- Coast protection
- Agricultural and fisheries
- Street cleanin not chargeable to highways
- Waste collection
- Waste disposal
- Trade waste
- Recycling
- Waste minimisation
- Climate change
- Cemerty cremation and mortuary
- Housing standards
- Health and safety
- Liscening - alcohol and enterainment liscencing; taxi liscencing
- Crime reduction
- Safety services

### Food Safety and Infection Conrol Functions (FSIC)

### Food Safety functions (FS)

- Regular hygiene inspections of food premises
- Routine inspection and testing of food samples
- Food hygiene courses for people handling food at work, in local communities and ethnic minorities, including training to implement Hazard Analysis Critical Control Points (HACCP)
- Food safety advice on planning applications related to food premises
- Production of advisory literature and training materials for food handlers
- The cost of licensing butchers’ shops and other premises identified under the Pennington report on food hygiene
- The operation of a ‘good hygiene’ certificate scheme
- Investigations into food poisoning outbreaks and food-borne illness

Record any licence fee income on line 230.(1)

### Animal Public Health and Infectious Disease Control Functions (Infection Control) (IC)

- Animal welfare
- Dog control
- Cesspool emptying
- Contributions to sewerage schemes
- Temporary caravan sites
- Checking conditions at travellers’ sites
- Health education activity, eg leaflets, exhibitions, etc
- Infectious disease

(1)

## Environmental and Regulatory expenditure


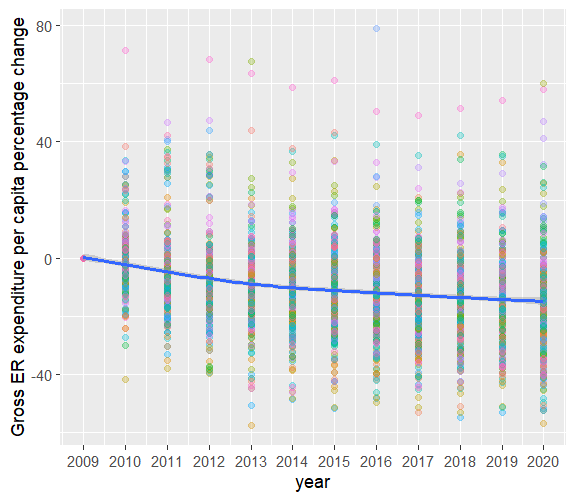


*Figure S1* Percent change in Environmental and Regulatory service expenditure annually by local authority, relative to 2009. Each circle corresponds to an individual local authority, blue line represents the average change each year.


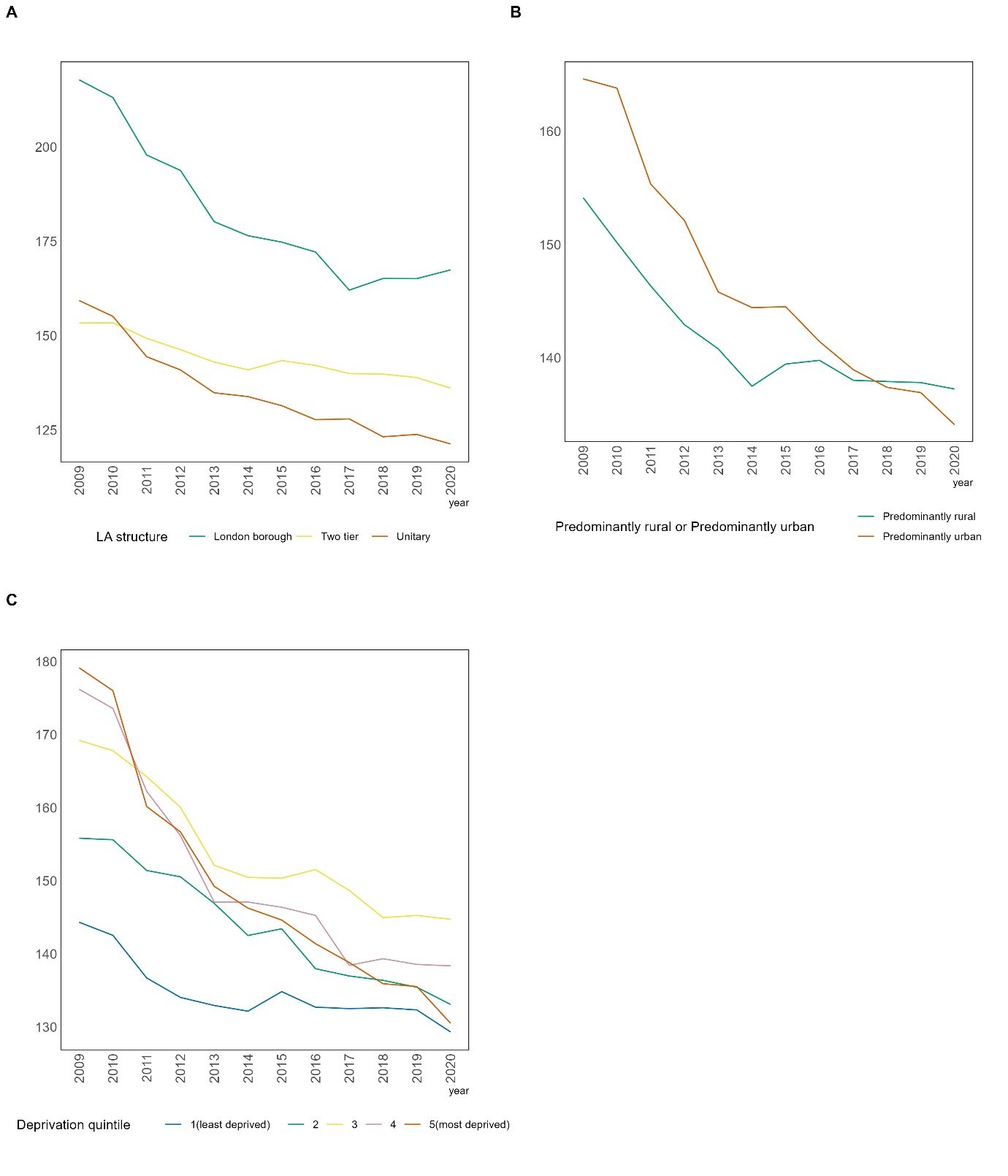


*Figure S2* Average Environmental and Regulatory expenditure (£) per capita between 2009 and 2020, stratified by local authority structure, rural and urban and by deprivation quintile.

## Food Safety + Infection Control expenditure


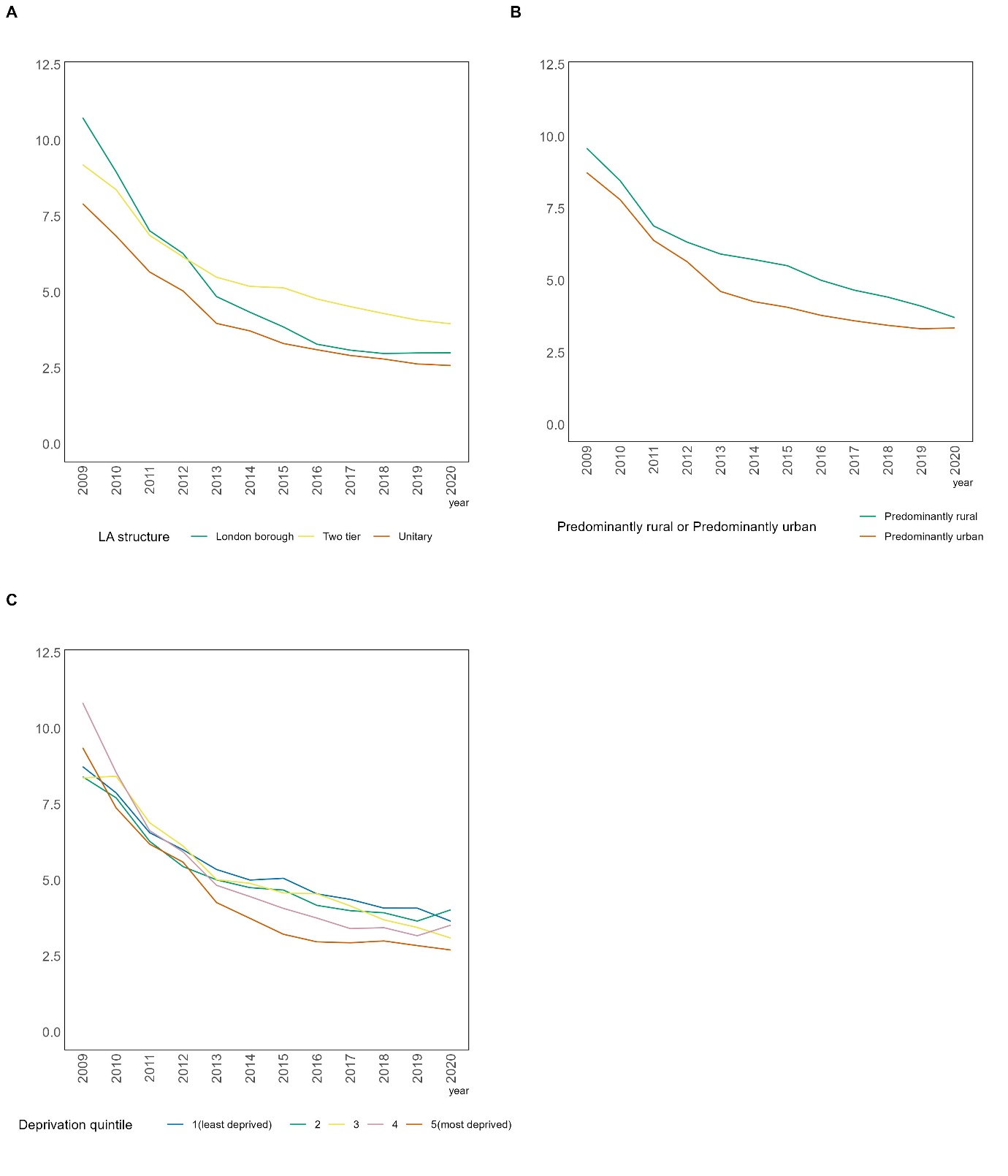


*Figure S3* Average Food Safety and Infection Control (£) per capita between 2009 and 2020, stratified by local authority structure, rural and urban and by deprivation quintile.

## Missing data

*Note: The following local authorities provided no service expenditure data for the specified years; Havant 2017, East Hampshire 2017, Newark and Sherwood 2012,2013 and Folkestone Hythe for 2017 and 2018.*

Table S1: The percentage of data reported as zero by service line from 2009-2020

| Environmental and Regulatory Sub-streams | % of observations reported as 0 |
| --- | --- |
| Agricultural and fisheries services | 54.9 |
| Animal and public health infectious disease control (Infection Control) | 7.4 |
| Cctv | 29.9 |
| Cemetery cremation and mortuary services | 7.8 |
| Climate change costs | 59.7 |
| Coast protection | 76.6 |
| Crime reduction | 11.6 |
| Defences against flooding | 37.9 |
| Environmental protection noise and nuisance | 8.9 |
| Food safety | 6.3 |
| Health and safety | 28.9 |
| Housing standards | 54.7 |
| Land drainage and related work | 44.4 |
| Licensing alcohol and entertainment licensing taxi licensing | 3.2 |
| Pest control | 19.2 |
| Port health | 93.6 |
| Public conveniences | 10.7 |
| Recycling | 7.5 |
| Safety services | 29.9 |
| Street cleansing not chargeable to highways | 1.1 |
| Trade waste | 33.6 |
| Trading standards | 0.5 |
| Waste collection | 0.5 |
| Waste disposal | 0.1 |
| Waste minimisation | 37.3 |
| Water safety | 74 |
| Aggregated Variable |  |
| Food safety and Infection Control Services | 1.2 |

*Figure S4* Flow diagram displaying data exclusion logic model for excluding local authorities from statistical analysis.

Table S2: Local authorities with missing data that were removed or included for multiple imputation.

| Excluded Local authorities | | Local authorities carried forward for multiple imputation | |
| --- | --- | --- | --- |
| LA code | LA name | LA code | LA name |
| E06000016 | Leicester | E06000042 | Milton Keynes |
| E06000035 | the Medway towns | E07000086 | Eastleigh |
| E06000045 | Southampton | E07000111 | Sevenoaks |
| E06000056 | Central Bedfordshire | E07000116 | Tunbridge wells |
| E08000008 | Tameside | E07000166 | Richmond shire |
| E09000011 | Greenwich | E07000177 | Cherwell |
| E06000007 | Warrington | E09000008 | Croydon |
| E06000012 | Northeast Lincolnshire | E09000030 | Tower hamlets |
| E07000066 | Basildon |  |  |

Density plot shows the observed values (blue), and estimated values from multiple imputation (red).


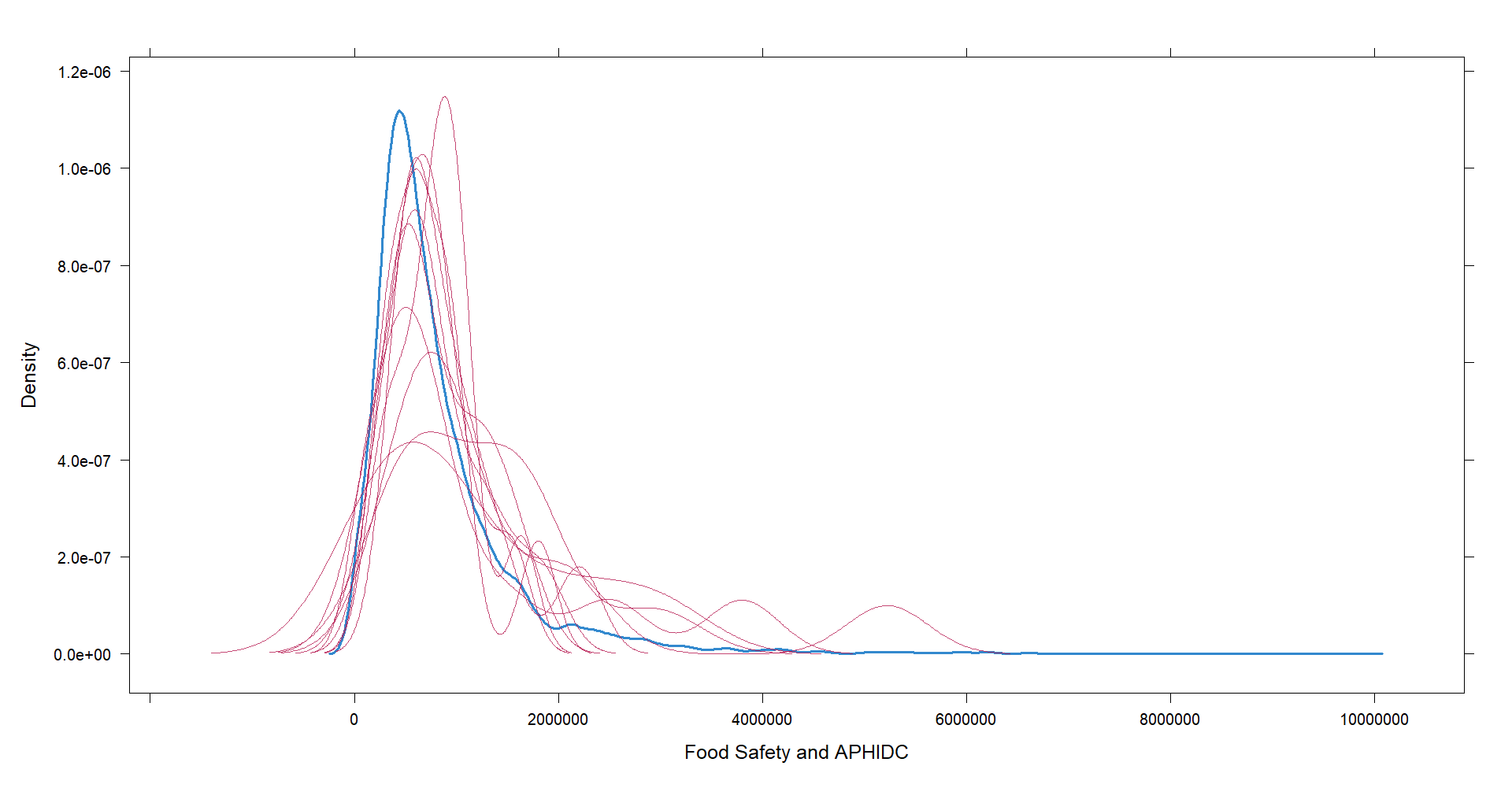


*Figure S5* Density plot showing observed values (blue) and estimated values (red) from multiple imputation.

## Sensitivity analysis

##### Sensitivity analysis: GEE results for case wise deletion

Table S3: Estimates of annual percentage change in Food Safety and Infection Control expenditure per capita from generalised estimating equation models, between 2009/10 and 2020/21 using case wise deletion (removal of all local authorities with reports of 0 for any year/ service)

| Variable | Annual percentage change in Food Safety +Infection Control expenditure per capita (95% Confidence Intervals) | Annual percentage change in Food Safety + Infection Control expenditure per capita as a share of ER (95% Confidence Intervals) |
| --- | --- | --- |
| 1 (least deprived) | -7.7(-9.3, -6.1) | -6.4 (-8, -4.8) |
| 2 | -9.2, (-12.3, -5.9) | -7.9 (-11.1, -4.6) |
| 3 | -6.2 (-8.8, -3.6) | -4.6 (-7.1, -1.9) |
| 4 | -9.1(-13.3, -4.6) | -6.1( -10.6, -1.3) |
| 5 (most deprived) | -24.1 (-36, -10) | -13.7 (-21, -5.8) |
| Two tier | -7.8(-9.3, -6.2) | 6.4 (-8.0, -4.8) |
| Unitary | -9.3 (-11.6, -6.9) | -8.8 (-11.2, -6.4) |
| London Borough | -10.7(-17.1, -3.8) | -7.8 (-14, -1.1) |
| Population density | -7.8 (-9.5, -6.1) | -6.4 (-8.1, -4.6) |

### Reference list

1. General fund revenue account outturn: specific guidance notes - GOV.UK [Internet]. [cited 2023 Jul 21]. Available from: https://www.gov.uk/government/publications/general-fund-revenue-account-outturn/general-fund-revenue-account-outturn-specific-guidance-notes#ro5-cultural-environmental-regulatory-and-planning-services
